# A Text-Book of Bacteriology

**Published:** 1891-11

**Authors:** 


A Text-Book of Bacteriology. By Carl Fraenkel, M. D., Professor of Hygiene, University of Königsberg. Third edition; translated and edited by J. H. Lindsley, M. D., Professor of Pathology and Bacteriology, Medical Department of the University of Vermont; Demonstrator of Pathology and Bacteriology, New York Post-Graduate Medical School and Hospital, etc., etc. Octavo, 380 pages. Extra muslin, $3.75. New York: William Wood & Company.

The scope of this work on Bacteriology is broader than the one next reviewed, in that it is intended for beginners in bacteriology. The names of its author and translator are sufficient to stamp it as authoritative, and, in a new science like bacteriology, no information is so good as that which comes from the fountain-head. To all American students who have worked under Professor Fraenkel, this translation will be joyfully welcomed.

The first few chapters are devoted to the methods of investigation, and so thoroughly is each step explained, that the student need have but patience and the materials to follow out the instructions. The development of the microscope to keep pace with the requirements of this subject, the points of superiority of the various stains and their formulæ, the methods of breeding, preparation of the different cultures, sterilization, etc., etc., are all carefully and minutely described.

Chapter IV. is devoted to questions pertaining to the bacteria themselves—their powers of resistance, mode of transmission,

Metchnikoff's phagocytic theory, of which the author seems to be somewhat in doubt, Koch's rules for the determination of pathogenic bacteria, theories of immunity, etc.

Chapter V. treats of the non-pathogenic bacteria, and Chapter VI. of the pathogenic, or those bacteria which, by their vital action, produce excretions injurious to the bodies of men and animals. In the discussion of these forms, the author has omitted those theories not founded on fact or observation, and treats only of things which bear strict criticism. Some thirty-two varieties of bacteria are treated under the head of pathogenic, and we have but one criticism to offer, namely, that no plates or illustrations are found in the whole treatise. We deem this a serious mistake, for, in all text-books, the illustrations—if they be accurate—convey more information than the text. The mold and yeast fungi are considered rather too briefly in the appendix. One thing is apparent in the perusal of the work, and that is the vast debt which bacteriology owes to its founder—Koch.

The typographical work is by William Wood & Company, and in succeeding editions we hope they will insist upon having well-executed plates, such as one would expect from the author.

